# Supplementary material for: Evidence supported by Mendelian randomization: impact on inflammatory factors in knee osteoarthritis
Source: Front Med (Lausanne). 2024 May 28;11:1382836. doi: 10.3389/fmed.2024.1382836 (PMC11165061; doi:10.3389/fmed.2024.1382836)
Supplement: Supplementary file 1 [file Data_Sheet_1.ZIP › data/GCST90274810_5e6/810.leaveoneout.pdf]

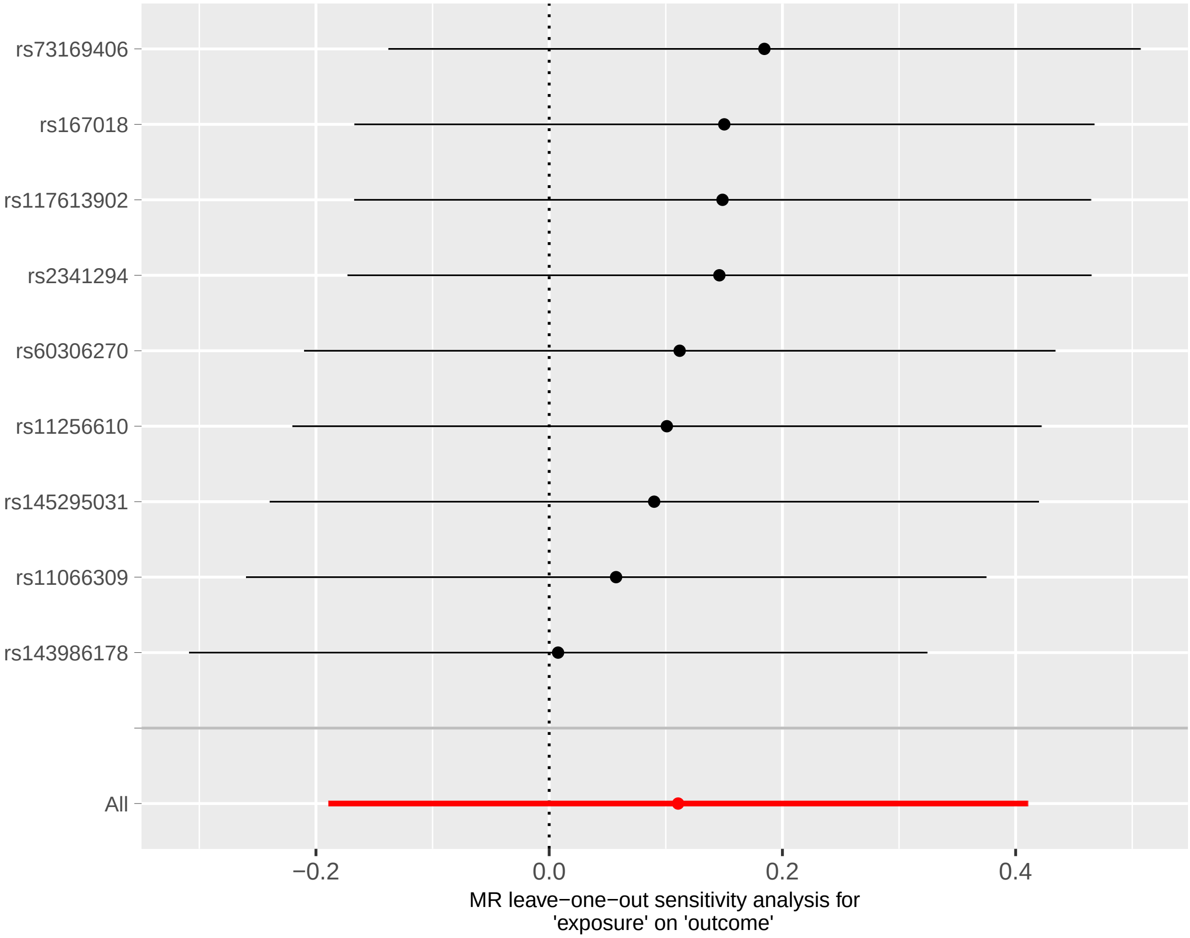

rs73169406
rs167018
rs117613902
rs2341294
rs60306270
rs11256610
rs145295031
rs11066309
rs143986178
All
−0.2
0.0
0.2
0.4
MR leave−one−out sensitivity analysis for
'exposure' on 'outcome'
